# The H2-A Class II molecule α/β-chain *cis-*mismatch severely affects cell surface expression, selection of conventional CD4^+^ T cells and protection against TB infection

**DOI:** 10.3389/fimmu.2023.1183614

**Published:** 2023-06-22

**Authors:** Nadezhda Logunova, Marina Kapina, Elena Kondratieva, Alexander Apt

**Affiliations:** Laboratory for Immunogenetics, Central Research TB Institute, Moscow, Russia

**Keywords:** H2-recombinant mice, MHC-II, recombination hot spot, T-cell selection, class II expression, tuberculosis infection

## Abstract

**Introduction:**

To dissect the role of the part of the *H2* complex comprised of the MHC-II genes in the control of tuberculosis (TB) infection, we previously established a panel of recombinant congenic mouse strains bearing different segments of the *H2* ^j^ haplotype on the B6 (*H2* ^b^) genetic background. Fine genetic mapping, gene sequencing and assessment of TB phenotypes resulted in identification of the *H2-Ab* gene as a major factor of TB control.

**Methods:**

We further narrowed the MHC-II *H2* ^j^ interval by spotting a new recombination event, sequencing newly established DNA configuration and establishing a mouse strain B6.I-103 in which *j/b* recombination occurred within the coding sequence of the *H2-Ab* gene.

**Results:**

Unexpectedly, a novel *H2-Aα* ^b^/*A*β^j^E^0^ haplotype provided exclusively high susceptibility to TB challenge. Immunologic analysis revealed an altered CD4^+^ T-cell selection and maintenance in B6.I-103 mice, as well as seriously impaired expression of the H2-Aα^b^/Aβ^j^ molecule on the surface of antigen presenting cells. Unlike previously reported cases of Class II malfunctioning, the defective phenotype arose not from strong structural mutations, but from regular recombination events within the MHC-II recombination hot spot region.

**Discussion:**

Our findings provide evidence that Class II α/β-chain *cis*-allelic mismatches created by regular genetic recombination may severely affect immune system functioning. This issue is discussed in the context of the MHC evolution.

## Introduction

Major histocompatibility complex Class II (MHC-II) molecules, expressed as α/β-chain heterodimers on the surface of immune cells (major populations are thymus epithelial cells, dendritic cells, activated macrophages and B-lymphocytes), present antigenic peptides to CD4^+^ T-lymphocytes and play a key regulatory role in adaptive immune responses (1, 2). In addition to an endless list of publications on MHC-II expression and CD4^+^ T-cell selection/maintenance summarized in books on MHC biology, direct evidence quantitatively linking the level of MHC-II expression with CD4^+^ T-cell functioning is accumulating, albeit slowly. Thus, it was demonstrated that mutant mice with ∼8-fold reduced H2-I-A expression (I-A^12%^ mice) possess a significantly decreased CD4^+^ T-cell subset, the phenotype accompanied by autoimmune-like reactivity and less efficient response against the intracellular bacterium, *Listeria monocytogenes* (3). Recently, a novel mutation (eight-base deletion in *h2aα* mRNA) resulting in an amino acid deletion in the H2-Aα molecule and significant numerical and functional reductions in the CD4^+^ T-cell population was identified (4). Given that a total abrogation of MHC-II expression due to either KO mutations in Class-II-encoding genes themselves, or dysfunction of key genetic regulators of their expression, lead to a well-known severe immunodeficiency called type II bare lymphocyte syndrome (5, 6), there is no doubt that seriously impaired MHC-II expression leads to profound CD4-associated immune aberrations.

A plethora of seminal studies performed about 20-30 years ago provided a detailed picture of MHC-II α/β chain assembly, the role of the invariant chain and antigenic peptides in this process and evaluated the relative stability of complexes formed by different allelic variants (7–11). However, no data have been reported concerning natural variations in the level of MHC-II expression dependent exclusively upon allelic polymorphisms in α- and β-chains and their combinations in different inbred and *H2*-recombinant congenic mouse strains. Most likely, this gap in the detailed knowledge of MHC biology is due to: (i) substantial differences in affinity and cross-reactivity of different anti-MHC-II antibodies used by different researchers to evaluate the expression levels, and (ii) extremely polygenic regulation of the expression phenotype itself. To overcome this latter obstacle, one should create mouse strains with very subtle Class II region differences on strictly identical genetic backgrounds.

Meanwhile, the other side of the equation, that is, variation in sizes of the CD4^+^ T-cell subset and CD4: CD8 ratios in inbred and *H2*-congenic mice bearing different *H2* haplotypes, has been relatively well studied, and many examples were published (Ref (12–14) [summarized in **Table S1** in Ref. (15)]. In an attempt to perform fine mapping and identify genes involved in the control of tuberculosis (TB) infection, we developed a large panel of *H2*-congenic mouse strains bearing small segments of the “TB-susceptible” *H2* ^j^ haplotype on the background of “TB-resistant” B6 (*H2* ^b^) mice (16). Within this panel, we developed and described the *I-E*-negative congenic strain B6.I-9.3 with profoundly altered selection of CD4^+^ T-cells in the context of their only classical Class II molecule I-A^j^. In these mice, despite the absence of mutations interfering with normal transcription and translation of the H2-Aα- and H2-Aβ-encoding genes, the ability of the A-αj/A-βj/E0 allelic combination to select in the thymus and maintain in the periphery normal numbers of conventional CD4^+^ T-cells was significantly impaired. In addition, the CD4^+^ T-cells themselves displayed a considerably narrowed repertoire of the TCR CDR3 region (15). Mice carrying the novel *A-α^j^/A-β^j^/E^0^* haplotype demonstrated an increased level of susceptibility to TB infection. Apparently, this was the first reported example of a major functional Class-II defect that had arisen not from a strong structural mutation or artificial genetic manipulation, but from regular recombination events within the MHC-II recombination hot spot region. Despite its functional defectiveness regarding TB protection, the A-α^j^/A-β^j^ dimer was expressed on the B-cell surface at levels similar to those of its A-α^b^/A-β^b^ counterpart, indicating redundancy in regulation of the “MHC-II expression – CD4^+^ T-cell selection” complex phenotype. In this regard, it is worth mentioning that in I-A^12%^ mice detectable numbers of CD4^+^ T-cells in lymphoid organs were present (3), suggesting that even profoundly reduced MHC-II context is sufficient for CD4^+^ cell selection and maintenance, albeit at lower levels.

The next step of our study was mating B.I-9.3 mice with prototype B6 mice in an attempt to further dissect the *H2-A*-occupied chromosomal region, anticipating that the presence of recombination hot spots would facilitate the appearance of recombinant alleles despite the very short genetic distance under study. Among developed (B6-9.3 x B6) F2 hybrids, the mouse number 103 provided the PCR product for the Aβ-chain-encoding sequence consistent with a recombination event between *j* and *b* alleles that led to almost entire replacement of *Aβ* ^b^ with *Aβ* ^j^ genetic material (see Material and Methods), creating a novel allelic configuration – *H2-Aα^b^/Aβ^j^* – of the only expressed Class II molecule. The progeny of this mouse was used to establish a new B6.I - 9.3.103 (hereafter – B6.I-103) congenic strain. In the present study, we describe hyper-susceptibility of B6.I-103 mice to TB infection in the context of altered CD4^+^ T-cell selection and maintenance, as well as severely impaired surface expression of the H2-Aα^b^/Aβ^j^ molecule on B-cells. Our findings provide evidence that Class II α**/**β-chain allelic mismatches created by regular genetic recombination events may be sufficient to severely impair immune system functioning.

## Materials and methods

### Mice and genotyping

Mice of inbred strains C57BL/6JCit (B6, *H2-A^b^*) and *H2*-recombinant congenic strain B6.I-9.3.19.8 (B6.I-9.3, *H2-A^j^*) were bred and maintained under conventional, non-specific pathogen-free (non-SPF) conditions at the Animal Facilities of the Central Institute for Tuberculosis in accordance with the guidelines from Russian Ministry of Health #755 and under the NIH Office of Laboratory Animal Welfare Assurance #A5502-11. Mice aged 8-12wk in the beginning of experiments were used. For genotyping, DNA was extracted using Wizard Genomic DNA Purification Kit (Promega, Madison, WI, USA) from mouse tail tips, and PCR was performed with primers for microsatellite markers (SSLP) D17Mit21, D17Mit 22 and D17Mit103 located within the region 34,4-34,552 Mb of the Chr. 17 (see the Jackson Laboratory database www.jax.org).

### Infection

*Mycobacterium tuberculosis* strain H37Rv (sub-strain Pasteur) was grown, stored and animals infected with 100 CFU of H37Rv using aerosol exposure chamber (Glas-Col, Terre Haute, IN, USA) as previously described (17). To determine mycobacterial counts in organs, lungs and spleens from individual mice were isolated in sterile conditions and homogenized in 2 ml of saline; series of ten-fold dilutions were prepared and plated (50μl per dish) on Petri dishes with Dubos agar (Difco, Sparks, MD, USA). Colonies were counted after 21 days of incubation at 37°С. Survival time was monitored daily starting week 3 post-infection.

### Congenic strain B6.I-103 generation

The ancestor of the B6.I-103 strain was obtained from F2 progeny of (B6 x B6.I-9.3) F1 intercross during selection of progenitors carrying recombination(s) between the markers D17Mit21 and D17Mit22. The recombinant mouse number 103 was mated with the B6 partner and their progeny intercrossed for further genotyping and selection of homozygous carriers of the novel recombinant haplotype *H2-A* ^b/j^.

### Mapping of recombination spot in B6.I-103 mice

The region between the markers D17Mit21 and 22 contains the following genes: H2-Ab1, H2-Aa, H2-Eb1 and H2-Eb2. To clone the H2-Ab1 and H2-Aa genes, RNA was extracted from spleens using SV Total RNA Isolation System (Promega, Madison, WI, USA) and treated with DNase I (AMPD1,Sigma-Aldrich, St.Louis, USA). cDNA was synthesized with oligo-dT18 primers (Thermo Fisher Scientific, Inc) and M-MLV reverse transcriptase (Promega, Madison, WI, USA). Primer sequences for cloning were selected from the Ensembl database (version GRCm39) for the H2-Ab1, H2-Aa genes of C57BL/6 mouse strain. 5’ forward primers ended up at the ATG start codon; reverse primers started at the TGA stop codon. Coding DNA was amplified using Advantage GC Genomic LA Polymerase (Clontech, Takara Bio, USA, Inc). PCR products were extracted from gels with Cleanup Mini Set (Evrogen, Moscow, Russia) and cloned in the PCR-Script Amp Cloning vector using the PCR-Script™ Amp Cloning Kit (Agilent (Stratagene, Santa Clara, CA,USA) or pAL-TA (Evrogen, Moscow, Russia) after 3 preliminary cycles of PCR products amplification with Taq-polymerase (Helicon, Moscow, Russia). Four-five positive clones were sequenced for each gene.

### Lung cell preparations and flow cytometry

Single cell suspensions were prepared from individual mouse. Infected B6, B6.I-9.3 and B6.I-103 mice were euthanized by injection of the thiopental overdose, and lung cell suspensions were prepared using the methods described earlier (18). Briefly, blood vessels were washed out *via* cut *vena cava* and repeated broncho-alveolar lavage was performed using 0.02% EDTA-PBS with antibiotics. Lung tissue was sliced into 1-2 mm^3^ pieces and incubated at 37^0^C for 90 min in RPMI-1640 (HyClone, Logan, UT, USA) containing 5% FCS (GE HealthCare, Chicago, IL, USA), penicillin-streptomycin, 10mM HEPES (HyClone, Logan, UT, USA), 200 U/ml collagenase and 50 U/ml DNase-I (Sigma-Aldrich, St.Louis, USA). Single cell suspensions were obtained by vigorous pipetting. Lung cells were washed twice in HBSS containing 2% FCS and antibiotics. Suspensions of thymus, spleen, and lymph node cells were prepared using routine homogenization.

Cells were incubated 5 min at 37^0^С with анти-CD16/CD32 mAbs (clone 93, Biolegend, San Diego, CA, USA) for Fc-receptors blocking and stained with the following antibodies against surface markers for FACS analysis: CD3-APC (clone 17A2), CD4-PerCP(or BV421 clone GK1.5) (clone GK1.5), CD8-APC (or PE or PerCP or A488; clone 53-6.7), CD19-PE (or BV510 clone 6D5), B220-APC (clone RA3-6B2), CD62L-PE(or APC) (clone Mel-14), CD44-FITC (clone IM7), CD11b-biotin (clone M1/70), CD11c-Alexa488 (or APC) (clone N418), Ly6G-PE (clone 1A8), streptavidin-PerCP (Biolegend); CD3-FITC (clone 145-2C11), or APC-H7 BD (clone GK1.5) (BD Pharmingen)

Anti-MHC-II H2-A-Alexa Fluor 488 (clone 10-2-16) mAbs cross-reactive with the H2-A^j^ β-chain was a kind gift of T. Golovkina, University of Chicago.

***Intracellular staining*** for the Treg marker Foxp3-PE (clone FJK-16s, eBiosciences, San Diego, CA, USA) performed according to the manufacturer’s protocol for Foxp3/Transcription Factor Staining Buffer Set (eBioscience). Intracellular staining of MHC class II molecules was performed according the same protocol with either mAb 10-2-16 or rabbit polyclonal antibodies to the cytoplasmic tails of MHC-II. Polyclonal antibodies to the MHC class II cytoplasmic tails were kindly provided by A. Rudensky (Memorial Sloan Kettering Cancer Center, New York).

For intracellular cytokine staining, 1.5 × 10^6^ cells were cultured overnight in the presence of mycobacteria cultural filtrate (10μg/ml) and GolgiPlug (1 μl/ml; BD Biosciences) for the last 12 hours. Then cells were stained with anti-CD4 and CD8 mAbs, followed by intracellular staining using the Cytofix/Cytoperm kit (BD Biosciences) with IFNγ-APC mAb (clone XMG1.2), and IL-17-PerCP mAb (clone TC11-18H10.1, both BD Biosciences). Data were acquired using BD FACSCalibur or BD FACSCanto II flow cytometers and analyzed using FlowJo software (Tree Star).

### Mixed lymphocyte reaction

CD4^+^ T-cells were isolated from spleens of B6 and B6.I-103 mice and purified using magnetic micro beads negative selection kit (Miltenyi Biotec, Bergisch Gladbach, Germany) with ~95% purity. T-cells (10^5^ per well) were cultured for 6 days at 37^0^C in supplemented RPMI 1640 medium (10% FCS, 10 mM HEPES, 4 mM L-glutamine, 5 x 10^-5^ M 2-ME, vitamins, piruvate, non-essential amino acids and antibiotics (all components – HiClone, Logan, UT, USA) in 96-well plates (Costar, Badhoevedorp, The Netherlands) in triplicates in the presence of mitomycin C-treated 3 x 10^5^ splenic APC from syngenic or allogenic donors. Cultures were pulsed with 0.5 µCi/well [^3^H]-thymidine for the last 18 h. The label uptake was measured in a liquid scintillation counter (Wallac, Finland) after harvesting the well’s contents onto fiberglass filters using a semi-automatic cell harvester (Scatron, Norway). Results were calculated using the following formula:


$$\begin{array}{l} \text{Response coefficient}\left(\text{RC}\right)=\Delta \text{cpm}(\text{T}-{\text{cells}}_{103}\text{vs}{\text{. APC}}_{\text{B}6}\\ -\text{T}-{\text{cells}}_{103}\text{vs}{\text{. APC}}_{103}):\\ \Delta \text{cpm}(\text{T}-{\text{cells}}_{\text{B}6}\text{vs}{\text{. APC}}_{103}-\text{T}-{\text{cells}}_{\text{B}6}\text{vs}{\text{. APC}}_{\text{B}6}). \end{array}$$

To exclude possibility that B6 T-cells do not respond against B6.I-103 MHC-II-expressing cells due to their intrinsic inability to recognize the H2-A^j^ product, reaction of B6 CD4^+^ T-cells against B6.I-9.3 stimulating cells was performed as the control.

### LPS -induced B cell activation

Splenocytes were incubated with or without50 µg/ml *E. coli* LPS (Sigma-Aldrich, St.Louis, US) for 48h in supplemented RPMI-1640 containing 5% FCS collected, washed and stained for flow cytometry.

### Histology and immunohistochemistry

Left lungs were frozen in electronic cryotome (ThermoShandon^®^, Manchester, UK) in the regimen of –60^0^C to –20^0^C temperature gradient. Serial 8µm-thick sections were made across the widest area. Sections were fixed with ice-cold acetone and stained with hematoxylin and eosin. Immunohistochemical assessment of CD4^+^ T-cells was performed on lung cryosections fixed with ice-cold acetone and blocked with 10% normal goat serum using peroxidase-conjugated anti-CD4 mAbs (clone H129.19, BD-PharMingen, San Diego, CA), with hematoxylin counterstaining. Slides were examined by the experienced mouse pathologist (EK) and photographed using Axioskop40 microscope and AxioCamMRc5 camera (Carl Zeiss, Berlin, Germany).

***Statistical analysis*** was performed using GraphPad Prism 5.0. Representative data from two to four independent experiments are displayed. Survival curves were analyzed by the log-rank test. For comparison of cell population sizes and ratios unpaired Students *t*-test was used. *P* < 0.05 was considered statistically significant.

## Results

### New recombinant mouse strain B6-103 and its TB-related phenotypes

**Figure 1:**
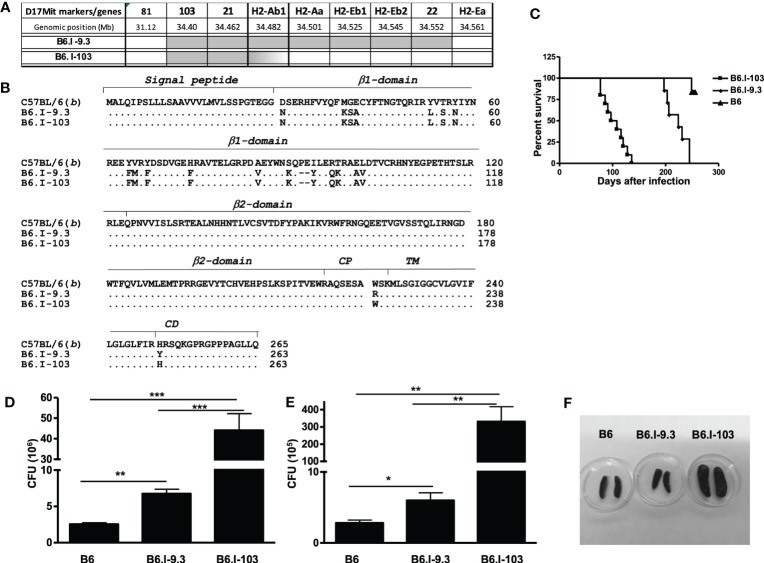
Genetic mapping of the recombination event that created novel *H2-Aα* ^b^/*Aβ* ^j^ *E^0^* Class II haplotype and TB susceptibility phenotypes of the B6-103 mouse strain. **(A)** – Chromosomal position of the recombination event between *H2-A* ^j^ (grey) and *H2-A* ^b^ (white) haplotypes identified in mouse #103 among (B6 x B6-9.3) F2 progeny and fixed in a novel B6-103 recombinant strain. Gene symbols and locations from the Ensemble Genome assembly GRCm39 (http://www.ensembl.org). **(B)** – Protein alignment of H2-Ab1 molecules in haplotypes under study in B6, B6-9.3 and B6-103 mice. Gene annotations are from UniProt Domain structure (http://www.uniprot.org): 1-27 – signal peptide; 28-122 – 1^st^ polymorphic domain; 123-216 – 2^nd^ conservative domain; 217-226 – connecting peptide (CP); 227-247 – trans-membrane domain (TM); 248-265 – cytoplasmic domain (CD). **(C)** – Mortality curves for B6, B6-9.3 and B6-103 mice , 10-12 mice per group in each of two similar experiments, log-rank test, *P<* 0.001. CFU mycobacterial counts in the lungs **(D)** and spleens **(E)** of infected mice, mean ± SD, 5 mice per group, one of two similar independent experiments is displayed, unpaired *t*-test. *, **, *** = *P<* 0.05, 0.01, 0.001, respectively. **(F)** – representative photos of spleens at week 8 post challenge.

Our previous genetic mapping within the MHC-II-occupied region resulted in establishing the B-9.3 mouse strain, narrowing the interval of Chr. 17 differentiating B.I-9.3 and B6 mice to the 30,900 – 34,335 Mb segment and prompting an investigation of which polymorphic markers might help to further genetically dissect the area (16). Approximately three hundred (B6 x B.I-9.3) F_2_ progeny were genotyped using a panel of additional polymorphic markers including four *D17Mit* SSLPs from the MGI-Mouse Genome Database (http://www.jax.org) and five classic and non-classic polymorphic MHC-II genes located within the *D17Mit81* – *h2ea* interval. In mouse number 103, j/b recombination occurred within the *h2ab* gene encoding the H2-Aβ chain of the H2-A molecule (**Figure 1A**). Sequencing of cDNA obtained from B6, B-9.3 and B-103 mice translated in the AA code demonstrated that the recombination event resulted in replacement of an entire polymorphic part of the H2^b^-Aβ1 domain with its H2^j^-counterpart. The cross-over point within the *H2^b^-Aβ* sequence is located somewhere between the D-encoding codon 102 in the β1 domain and the A-encoding codon 222 in the extracellular connecting peptide (CP) domain, including the whole non-polymorphic sequence encoding the β2 domain. Its more precise mapping was impossible since identical triplets between positions 102 – 222 are present in the *H2* ^b^ and *H2* ^j^ haplotypes (**Figure 1B**).

After establishing the B6.I-103 strain, we first assessed how further shortening of the *H2* ^j^-originated MHC-II segment influences TB susceptibility/severity, anticipating that the new strain would show either a susceptibility level similar to that of the ancestor B.I-9.3 strain (intermediate between I/St and B6 mice – Ref (16)), or a more resistant B6-like phenotype. To our surprise, B6.I-103 mice displayed an exclusively high level of TB susceptibility, exceeding that of all approximately 50 mouse strains previously tested in our lab, including the I/St ancestors. B6.I-103 mice succumbed to infection significantly earlier than B6.I-9.3 mice (**Figure 1C**), and mycobacterial multiplication in their primarily infected lungs and dissemination-infected spleens was significantly higher (**Figures 1D, E**). In addition, strong splenomegaly developed in B6.I-103 mice (**Figure 1F**).

We then assessed how susceptibility to infection was reflected in the general histopathology of the lung tissue at a more advanced stage of infection (8 weeks post challenge). As shown in **Figure S1**, in B6.I-103 mice inflamed areas of the lung were characterized by diffuse, non-structured pneumonia lacking any signs of granuloma formation, whereas in B6.I-9.3 mice circumscribed inflammatory foci clearly delineated from surrounding tissue and intra-bronchial space were readily observed. In B6 mice, granuloma formation was even more evident, including newly formed small granuloma comprised of a few centrally located macrophages surrounded by a thin layer of lymphocytes. This is in agreement with our previous results obtained in different mouse models of TB infection demonstrating that rapid and active granulomatous processes are evident in mice of genetically TB-resistant, but not in susceptible strains (19, 20).

**Figure 2:**
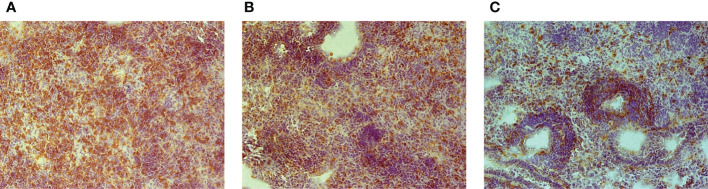
Early (3-wk) CD4^+^ T-cell infiltration of TB-infected lung tissue in B6 **(A)**, B6.I-9.3 **(B)** and B6-I.103 **(C)** mice. Peroxidase immune staining with anti-CD4 mAbs (brown) with hematoxylin counter-staining (blue). Representative microscopic fields of lung parenchyma. In B6 mice, layers of CD4^+^ cells entirely cover the field, being less solid in B6-9.3 and much more transparent in B6-103 mice, which allows seeing other cell types colored blue.

To determine whether an unusually rapid disease progression in B6.I-103 mice was accompanied by shifts in the lung tissue infiltration, we compared the lungs of B6, B6.I-9.3 and B6.I-103 mice with respect to the influx of CD4^+^ T-lymphocytes at the early phase of infection (week 3 post challenge). These cells are the major population of immune cells providing anti-TB defense (21, 22), and at this time point secondary mycobacterial seeding and activation of adaptive anti-TB immunity become evident in the mouse lungs (23, 24). Immune staining of lung cryo-sections for CD4^+^ T-cells revealed a remarkable difference between B6.I-103 and parental strains in the content of CD4^+^ T-cells in the lung tissue, including parenchyma and peribronchial/perivascular lymphocyte cuffs (**Figure 2**). In B6 mice, massive accumulation of CD4^+^ T-cells was evident even in parenchymal tissue, whereas in B6.I-9.3 and, especially in B6.I-103 mice, peribronchial lymphocyte cuffs which during TB comprise the majority of attracted T-cells, displayed many CD4^+^ T cell-free zones. The significant decrease in the CD4^+^ T-cell content in B6.I-9.3 compared to B6 mice reported earlier on the basis of flow cytometry evaluation (15), on the sections of lung tissue looked quite moderate compared to a marked deficiency of these cells in B6.I-103 mice.

### CD4^+^ T-cells in lymphoid organs of non-infected mice

**Figure 3:**
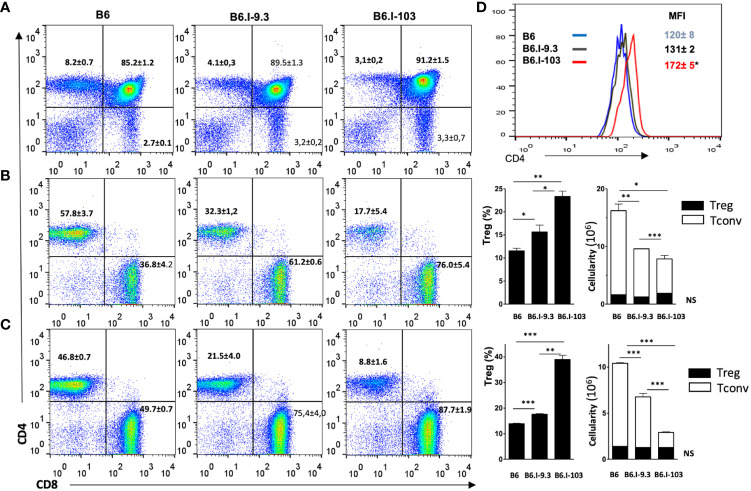
Analysis of T-cells in lymphoid organs of mice of the three strains. **(A)** – thymus; **(B)** – spleen; **(C)** – lymph nodes. B6.I-103 mice are deficient for CD4^+^ T cells in the thymus and periphery. The percentiles of CD4^+^ and CD8^+^ T-cells in gated CD3^+^ T-cell populations were assessed by flow cytometry. Representative data from one of three similar independent experiments including four mice each are displayed and total statistics for all measurements is provided as mean ± SD (see **Figure 1** legend for statistical explanations). **(B, C)** right panels – the content of CD4^+^Foxp3^+^ Treg and CD4^+^Foxp3^-^ Tconv cells. **(D)** – the expression level of the CD4 molecule on thymocytes is expressed as the magnitude of fluorescence intensity (MFI), asterisk = *P*< 0.05 between B6.I-103 and two ancestor mouse strains B6 and B6.I -9.3. NS, not significant. *, **, ***, **** = P < 0.05, 0.01, 0.001, 0.0001, respectively.

It was important to find out whether the CD4^+^ T-cell deficiency in B6.I-103 mice was intrinsic, as we previously reported for B6.I-9.3 mice (15), or depended upon rapidly developing TB infection. Statistical analysis performed by flow cytometry for cells from the thymus, spleen and lymph nodes clearly demonstrated a profound deficiency of the CD4^+^ subset in lymphoid organs of non-infected B6.I-103 mice (**Figures 3A–C**). We then evaluated if such a deficiency affected both conventional effector (Tconv) and regulatory (Treg) CD4^+^ T-cells. To this end, we quantitatively analyzed the ratios of FoxP3^-^ Tconv and FoxP3^+^ Treg CD4^+^ lymphocytes *vs*. CD8^+^ T-cells and the total numbers CD4^+^ T-lymphocytes in the organs. In all anatomical sites, we observed a clear decrease in the size of the Tconv population along the B6 → B.I-9.3 → B6.I-103 axis. In spleens (**Figure 3B**), the content of CD4^+^ Tconv cells comprised about 50% in B6.I-9.3 (CD4: CD8 ratio = 1: 2) and only about 25% in B6-103 mice (CD4: CD8 ratio = 1: 4), of that in B6 mice (CD4: CD8 ratio = ~1.5: 1.0). Analogous, but even more striking, differences were observed for lymph nodes (**Figure 3C**), in which the CD4: CD8 ratios were 1: 1, 1: 3 and 1: 10 in B6, B-9.3 and B6-103 mice, respectively. Even in the thymus, where the content of single-positive T-cells is normally low, the population of single-positive CD4^+^ cells was twice as big in B6 mice (**Figure 3A**). Statistical analysis displayed in the right panels of **Figures 3B, C** shows that the defects in CD4^+^ selection occurred only in Tconv cells. Whereas relative proportions of the Treg populations were apparently expanded in B6-9.3 and B6.I-103 mice (due to compensation for the Tconv deficiency?), no differences in Treg content per organ were observed. Interestingly, the expression level of the CD4 molecule on less effectively selected B6.I-103 thymocytes was significantly higher than on their B6 and B6.I-9.3 counterparts (**Figure 3D**), suggesting some compensatory reaction.

Taken together, these results clearly show that an impaired selection and maintenance of Tconv CD4^+^ T-cells in the lymphoid organs of B6.I-103 mice is independent of contact with mycobacteria, suggesting that their response to infection is intrinsically impaired.

### Immune cells in infected lungs

**Figure 4:**
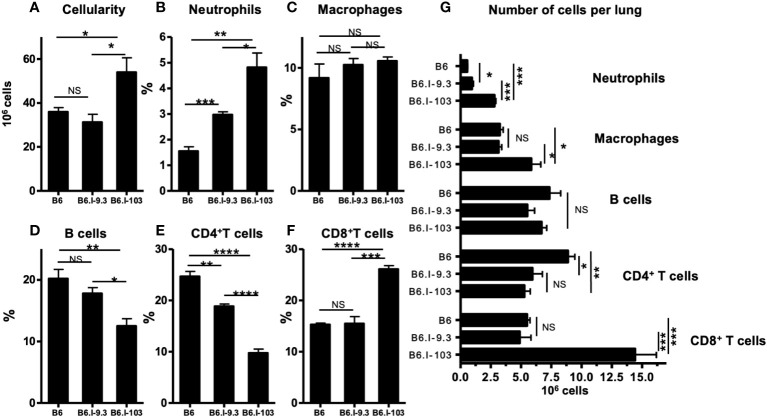
Flow cytometry analysis of *M. tuberculosis*-infected lungs. B6, B6.I-9.3 and B6.I-103 mice were infected with *M. tuberculosis* and their lung cells were stained with mAbs against B-cells (anti-CD19), CD4 and CD8 T cells (anti-CD4, anti-CD8), neutrophils (anti-Ly6G) and Treg cells (CD4^+^Foxp3^+^) at 40 days post challenge. In panels B-F marked with cell type headings, mean values ± SD (N = 4 per group in each of two similar experiments) are displayed as per cent of the total lung single cell suspension. *P* values were calculated using the unpaired *t*-test, for asterisk values see the legend for **Figure 3**. NS, not significant.

To assess how a more severe course of TB infection in CD4^+^ T-cell-deficient B6.I-103 mice influences immune processes in the lungs, we first compared the sizes of major immune cell population in infected mice. As shown in **Figure 4A**, infected lungs of B6.I-103 mice contained a significantly increased total number of cells compared to B6 and B6.I-9.3 mice, suggesting an elevated level of cellular infiltration. Relative content of all major immune cell populations (**Figures 4B, D**–**F**), except macrophages (**Figure 4C**), in B6.I-103 lungs significantly differed from that in mice of the ancestor strains. Besides CD4^+^ T-cells, whose diminished content was anticipated due to the results of experiments described above (**Figures 2**, **3**), the lungs of B6.I-103 mice contained fewer B-lymphocytes, but significantly more neutrophils and CD8^+^ T-cells. However, since the overall cellularity of infected B6.I-103 lungs was ~1.5-fold higher than that of mice of other two strains, a more reliable parameter would be the total number of cells per organ for each population. As shown in **Figure 4G**, a relative B-cell deficiency was not confirmed by the total counts, however, the deficiency in CD4^+^ T-cells remained evident. Two additional observations added to a general picture of TB pathogenesis on the background of CD4^+^ cell deficiency. First, a clear inversion of the CD4: CD8 ratio in the lymphoid system of B6.I-103 mice observed previously in healthy mice appeared also in the infected lungs, where the population of CD8^+^ T-cells expanded disproportionally post challenge. Second, in B6.I-103 mice the post-infection lung neutrophil influx was significantly higher than the level observed in the two other mouse strains.

Next, we evaluated production of IFN-γ and IL-17 by lung CD4^+^ T-cells, since the balance between these two antagonistic cytokines secreted by distinct populations of CD4^+^ T-cells is a key regulator of deleterious neutrophil inflammation in the tuberculous lung (25, 26). As shown in **Figures S2A, B**, at the advanced stage of TB the proportion of CD4^+^IFN-γ^+^ T-cells in the lungs of mice of all three strains was similar; however, since the total amount of CD4^+^ cells was significantly higher in B6 animals, their lungs contained significantly more CD4^+^ T-cells producing this cytokine. An even more impressive picture was observed for IL-17 production: both the relative proportion and total number of Th17 CD4^+^ cells was significantly higher in B6.I-103 lungs despite the general deficiency of CD4+ cells (**Figures S2C, D**).

### Expression of the H2-A products in B6.I-103 mice

**Figure 5:**
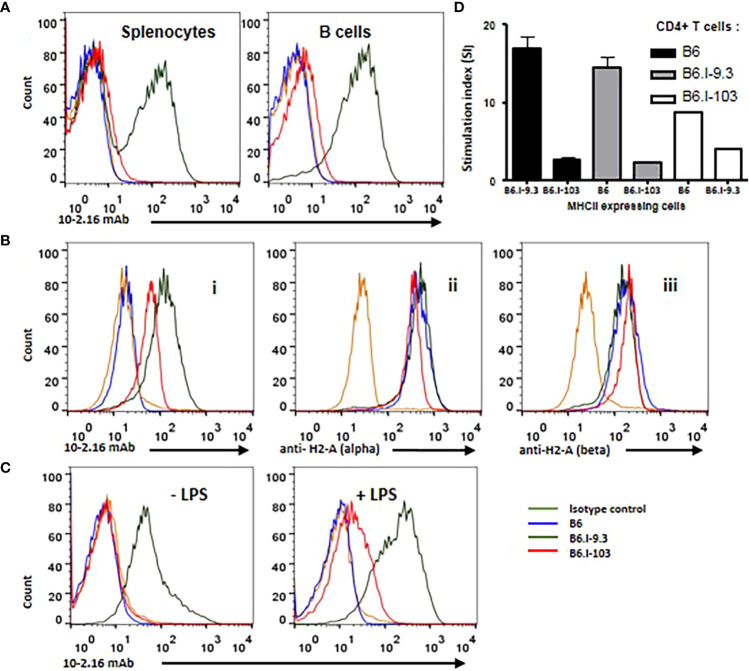
B-lymphocytes from B6.I-103 mice transcribe and translate H2-A α- and β-chains normally but cell surface expression of the α/β dimer is severely impaired. **(A)** – 10-2-16 anti-H2-A^j^ β-chain antibodies stain the surface of B6.I-9.3 but not B6.I-103 splenic B-cells. Flow cytometry histograms for total and B-lymphocyte-gated (double staining with anti-CD19) cell populations. **(B)** – Intracellular staining of gated CD19^+^ B-lymphocytes. Alexa488-lebeled anti-H2^j^ β-chain 10-2-16 mAbs (i); polyclonal rabbit antibodies to the cytoplasmic tail of H2-Aα chain (ii); (iii) polyclonal rabbit antibodies to the cytoplasmic tail of H2-Aβ chain. Polyclonal antibody staining was followed by polyclonal donkey anti-rabbit IgG (Fc) Alexa 647-labeled antibodies. **(C)** – LPS activation partly restores H2-A surface expression in B6.I-103 B-cells. Splenocytes were incubated *in vitro* with or without LPS for similar independent experiments are displayed. Mouse Ig2b served as the isotype control. See text for details.48h before double staining with anti-CD19 and 10-2-16 mAbs. Representative data from one of three similar experiments are displayed. **(D)** – The results of MLR response in indicated combinations of purified CD4^+^ T-cells against stimulating splenocytes are expressed as medium CPM ± SD of ^[3]^H-thymidine incorporation in triplicated each cell combinations. See M&M section for details.

We hypothesized that the explanation for the profound CD4^+^ Tconv cell deficiency in B6.I-103 mice described above would be a malfunction of their only Class II H2-A molecule responsible for selecting these cells in the thymus and maintaining them in the periphery. Thus, we compared the level of expression of hybrid H2-A^j/b^ molecules on antigen-presenting cells (APC) in these mice with corresponding products in their B6 (H2-A^b^) and B6.I-9.3 (H2-A^j^) counterparts. Working with Class II products of the *H2* ^j^ origin is always a challenge, since no commercially available specific anti-H2^j^ antibodies exist. Thus, in the first experimental series we stained splenocytes from mice of the three strains with the classic monoclonal antibody (mAb) 10-2-16 originally developed against the H2-A^k^ β-chain (27). These antibodies do not react with the H2^b^-A_β_, but cross-react with a few other allelic variants of the H2-A_β_-chain, including H2-A^j^ (Ref (28, 29). and our unpublished observations for H2^j^). As expected, these A_β_-chain-recognizing antibodies readily reacted with the H2-A^j^ product on the surface of B6.I-9.3 splenic B-cells and did not react with H2-A^b^ B-cells from B6 mice (**Figure 5A**). Surprisingly, there was only very weak staining of B6.I-103 cells, although the AA sequence of the H2-A_β_ chain extracellular domains in B6.I-9.3 and B6.I-103 mice is identical.

To assess whether defective expression of the H2-A dimer on the surface of B6.I-103 B-cells is possible to confirm in an experimental system independent of antibody staining, we performed experiments on allogenic recognition of B6 and B6.I-103 Class II products by purified CD4^+^ T-cells in the mixed lymphocyte cultures. It appeared that CD4^+^ T-cells from B6 mice demonstrate very similar and low (<200 cpm) ^[3]^H-thymidine incorporation when co-cultured with splenic APC from syngenic or B6.I-103 mice, whereas the level of B6.I-103 T-cell response against allogenic B6 APC was ~8-fold higher (**Figure 5D**). These results are consistent with an impaired expression of CD4^+^ T-cell-activating molecules on B6.I-103 APC.

Among several possible reasons for this discrepancy, either the synthesis of the H2-A_β_ chain or transportation of the H2-A dimer on the cell surface may be impaired in B6.I-103 mice. To distinguish these two possibilities, we performed intracellular staining of H2-A_β_ chains in B-cells from mice of the three strains. As shown in **Figure 5B** (left panel), inside B-cells, 10-2-16 antibodies stained H2-A^j^ β-chains of both B6.I-9.3 and B6.I-103 origin, again leaving unstained the H2-A^b^ product. However, some concerns remained about a full-blown synthesis of the Aβ chain in the B6.I-103 cells, given that its intracellular staining appeared to be somewhat lower than that in B6.I-9.3 cells (**Figure 5B**). Since 10-2-16 antibody reacts with the configuration-dependent antigenic determinant (which may differ in α/β H2-A^j/j^ and H2-A^j/b^ combinations providing different intensity of staining), we repeated the intracellular staining using polyclonal rabbit anti-mouse-H2-A antibodies specifically reacting with the cytoplasmic domains of either α- or β-chains (see Materials and Methods). As shown in **Figure 5B** (left panels), these antibodies stained Aα and Aβ intracellular chains in B6.I-9.3 and B6.I-103 B-cells similarly and with high intensity, indicating that the B6.I-103 defect affects the assembly and/or transportation of Class II dimers to the cell surface rather than biosynthesis of the monomers

This conclusion was further supported by the experiments on *in vitro* stimulation of B-cells from B6.I-103 mice with their classical activator LPS, whose capacity to up-regulate Class II expression at the transcriptional level is well demonstrated (30). As shown in **Figure 5C**, we found a readily detectable population of B6.I-103 B-cells expressing the H2-A molecule on their surface (10-2-16 antibody staining) after 48-h of incubation of the cells in the presence of LPS. In spite of the fact that the H2-A expression was markedly lower than that on the surface of B6.I-9.3 cells, these results clearly confirm that both the α- and β-chains of the H2-A molecules are available for assembly within B6.I-103 B-cells, and artificial stimulation of their synthesis increases the number of dimers capable of reaching the B-cell surface. This increased expression on the surface of activated B-cells, as well as the presence of small amounts of 10-2-16 mAb-stained B-cells in B6.I-103 spleens (**Figure 5A**), may explain why we observed a profound decrease, rather than a total lack, of CD4^+^ T-cell content in these mice. Certain numbers of activated cells should be present in the organs, especially in non-SPF mice receiving non-sterile food and water. Nevertheless, under normal conditions the H2-A surface expression, which is entirely dependent upon α/β-chain dimerization, is most likely is impaired due to the allelic *cis-*mismatch.

## Discussion

Usually, the most challenging part in the study of genetically impaired resistance to chronic infections is identification of the cellular and molecular mechanisms underlying the phenotypes observed. In the present work, a mechanistic explanation of the unusual susceptibility to and severity of TB infection in B6.I-103 mice is more or less obvious: a profoundly altered selection of CD4*^+^* T-cells in the context of a novel haplotype results in TB hyper-susceptibility (22). Thus, complicated discussions regarding this part of the present work can be avoided. It is worth mentioning only that alterations in TB-related immune reactivity in B6.I-103 mice in many instances are similar to those described earlier for TB-susceptible mice with genetic deficiencies related to macrophage (31, 32) and T-cell (24, 25) malfunctions. One impressive feature of TB pathogenesis in corresponding models is poorly controlled neutrophil inflammation on the background of unbalanced IL-17 production (31, 33), the phenotypes readily observed in this work (**Figures 4**, **5**). However, the genetic origin of this particular immune deficient phenotype is so peculiar that it deserves some discussion.

A severely impaired capacity to express H2-A α/β-chain dimers on the surface of APC in B6.I-103 mice (**Figure 5**) is not due to missense or nonsense mutations in the coding parts of the corresponding genes or stop codon appearance (**Figure 1B**). Moreover, their translation and transcription apparently were not altered, providing proteins readily recognized by antibodies inside B-cells (**Figure 5B**). It is likely that an impaired transportation to the outer layer of the cell membrane was due to a structural *cis*-mismatch between the chains interfering with their correct assembly. This is confirmed by the fact that the defect was rather quantitative than absolute, since artificial *in vitro* activation of B-cells from B6-103 mice with LPS resulted in the appearance of Class II dimers on their surface, albeit only on a proportion of cells (**Figure 5C**). Attempts of structural molecular modeling based upon DNA sequences of genes encoding α- and β-chains of the H2-A^b/j^ molecule, the approach applied in our earlier study (in Ref. 16 see Figures 7 and S1 and corresponding explanations) failed to provide a conclusive explanation for this mismatch.

This has left us with only a vague idea that some allelic variants of Class II molecules fail to form fully-functional dimer combinations, a proposal broadly discussed in the past. Thus, the concept of serious functional diversity between different allelic Class II α- and β combinations was firstly put forward and experimentally studied using F1 hybrids and cell line gene transfection approaches by C. Janeway, Jr. and R. Germain (34–36). Now it receives a direct *in vivo* confirmation from our work. Importantly, in this study, no gene manipulation approaches were applied, and a severe expression defect in the *H2-A* ^b/j^ allelic combination resulted from regular meiotic recombination events.

It has been known for decades that the Class II MHC chromosomal segment is exceptionally rich in meiotic recombination hot spots. This has been repeatedly demonstrated in mice (37, 38), cattle (39), non-human primates (40), humans (41, 42) and many other species (43). There is little doubt that a dramatically increased frequency of recombination between highly polymorphic, homologous and physically neighboring Class II genes is the driving force behind genetic diversity; a prerequisite for effective immune protection of host populations against different pathogens over evolutionary time. However, the results reported herein directly demonstrate that this genetic mechanism may also create recombinant variants severely affecting host-defending immune responses against infectious invasions. Despite our results, were obtained in a TB mouse model (and TB was not a selective factor in natural *Mus musculus* evolution), defective development of the CD4^+^ T-cell population most likely affects resistance to many different pathogens, including natural parasites of the mouse.

A very important evolutionary aspect of the proposed role of Class II recombination is its unprecedented frequency, at least in some allelic combinations, including *H2* ^b^ (44). Indeed, we identified the crossover B6.I-103 mouse as number 103 among (B6.I-9.3 x B6) F2 progeny (frequency = 10^-2^). The ancestor B6.I-9.3 mouse was obtained from one of the litters from [(B6 x I/St) F1 x B6] N6 cross (totally two crossovers within the 30.90- to 34.34-Mb interval of chromosome 17 were found among 212 mice, frequency = 10^-2^). The whole 15-y program of the Class II region recombination analysis in the *b*-*j* allelic combination included over 9,500 animals and provided more than 50 recombination events (frequency ~10^-2^). This convincingly demonstrates that the crossover frequency within the Class II region in this experimental setting comprises 1 per cent. This figure is many-fold higher than all estimations of the mutation rate for any mammalian gene, including the *H2-K* ^b^ allelic variant of the Class I gene present in B6 mice, which is believed to be the hottest mutation spot in the murine genome (45, 46). Mutations are commonly considered to be the main subject of natural selection, whilst the perception of recombination events is shifted towards generating genetic diversity rather than creating *de novo* variants that markedly improve or worsen fitness.

In this study, we experimentally demonstrated that due to its unprecedented frequency intra-Class II recombination may operate as a genetic super-power, at least within the immune system, providing serious functional changes in the molecules involved in immune responses. Our example, of course, illustrates only the negative part of the problem (appearance of an unsuccessful combination of the Class II molecule chains), and no firmly proven examples of recombination events improving immunity have been published so far. A quick look at the dates when the literature cited above were published shows that *MHC* evolution almost vanished – luckily not completely, see Ref (47) – from the list of the top problems of immunogenetics relatively long ago. This obviously did not happen because the problem has been successfully and comprehensively resolved. Thus, more recent publications providing a good body of evidence for the existence of *MHC*-non-restricted α/β-TCR T-cells (48–50), as well as an imaginative selection theory explaining the link between their existence, dependence upon Lck tyrosine kinase sequestration by TCR co-receptors and the capacity to signal *via* MHC molecules [summarized in Ref. (51)] strongly indicate that the story is far from being completed. We sincerely hope that our work will also revive interest in these fundamental aspects of immune system evolution.

## Data availability statement

The original contributions presented in the study are included in the article/**Supplementary Material**. Further inquiries can be directed to the corresponding author/s.

## Ethics statement

The animal study was reviewed and approved by The CIT IACUC, Central TB Research Institute, Moscow, Russia. IACUC protocols #2, 7, 8, 11 approved on March 6, 2022.

## Author contributions

NL, AA - conceptualization, NL, MK, EK - experimentation, data handling, statistics, NL, AA - wrote the manuscript. All authors contributed to the article and approved the submitted version.
