# Supplementary material for: The H2-A Class II molecule α/β-chain cis-mismatch severely affects cell surface expression, selection of conventional CD4+ T cells and protection against TB infection
Source: Front Immunol. 2023 Jun 22;14:1183614. doi: 10.3389/fimmu.2023.1183614 (PMC10324577; doi:10.3389/fimmu.2023.1183614)
Supplement: Supplementary file 1 [file DataSheet_1.pdf]

Supplementary figures

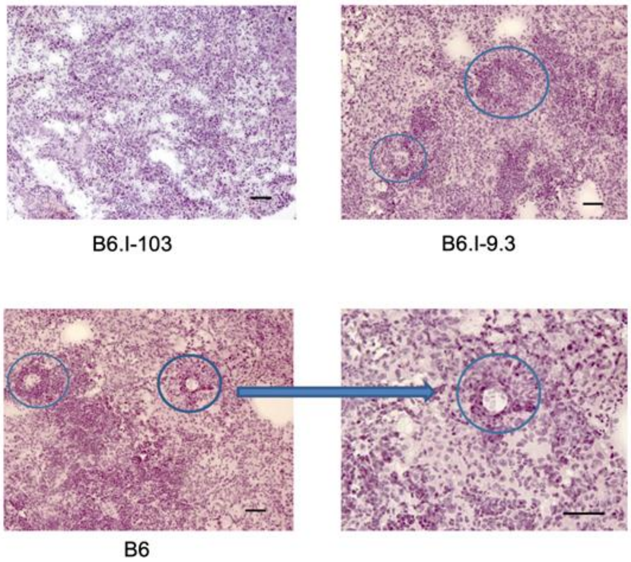

Figure S1. Diffuse, non-structured inflammation of the lung tissue in B6.I-103 mice and formation of circled granulomata (blue circles) in B6.I-9.3 and B6 mice. Formation of young small granuloma around macrophages is highlighted at a larger magnification for B6 mice (left bottom panel).

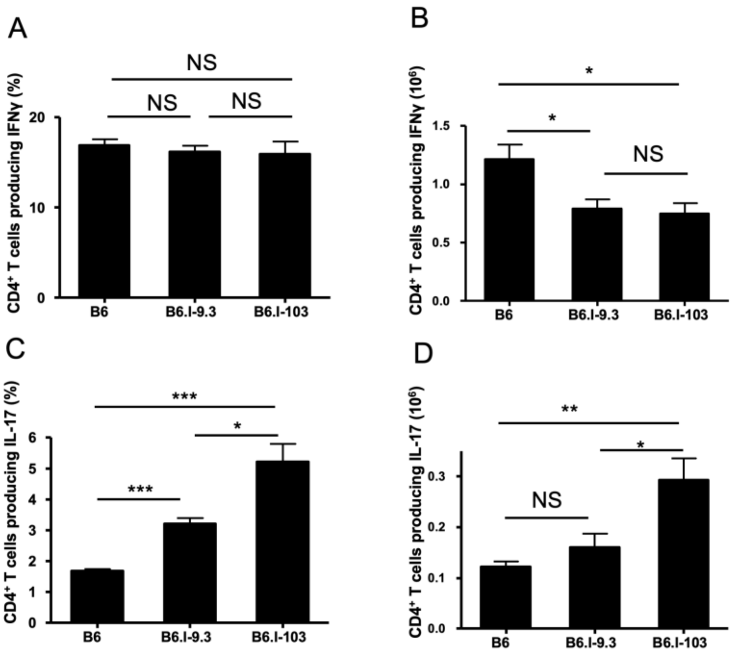

Figure S2. Reciprocal content of $CD4^+$ T-lymphocytes producing IFN-γ and IL-17 in B6 and B6.I-103 lungs.
